# Synthesis and Biological Evaluation of a Novel C8-Pyrrolobenzodiazepine (PBD) Adenosine Conjugate. A Study on the Role of the PBD Ring in the Biological Activity of PBD-Conjugates

**DOI:** 10.3390/molecules25051243

**Published:** 2020-03-10

**Authors:** Lindsay Ferguson, Sanjib Bhakta, Keith R. Fox, Geoff Wells, Federico Brucoli

**Affiliations:** 1 School of Science, University of the West of Scotland, Paisley, Scotland PA1 2BE, UK; 2 Department of Biological Sciences, Institute of Structural and Molecular Biology, Birkbeck, University of London, London WC1E 7HX, UK; 3 School of Biological Sciences, University of Southampton, Southampton SO17 1BJ, UK; 4 UCL School of Pharmacy, University College London, 29/39 Brunswick Square, London WC1N 1AX, UK; 5 Leicester School of Pharmacy, De Montfort University, Leicester LE1 9BH, UK

**Keywords:** C8-linked pyrrolobenzodiazepine (PBD) conjugates, DNA minor groove binding agents, DNase I footprinting, adenosine nucleoside, heterodimeric hybrids, High-throughput spot-culture growth inhibition assay, anti-mycobacterial compounds

## Abstract

Here we sought to evaluate the contribution of the PBD unit to the biological activity of PBD-conjugates and, to this end, an adenosine nucleoside was attached to the PBD A-ring C8 position. A convergent approach was successfully adopted for the synthesis of a novel C8-linked pyrrolo(2,1-*c*)(1,4)benzodiazepine(PBD)-adenosine(ADN) hybrid. The PBD and adenosine (ADN) moieties were synthesized separately and then linked through a pentynyl linker. To our knowledge, this is the first report of a PBD connected to a nucleoside. Surprisingly, the compound showed no cytotoxicity against murine cells and was inactive against *Mycobacterium aurum* and *M. bovis* strains and did not bind to guanine-containing DNA sequences, as shown by DNase I footprinting experiments. Molecular dynamics simulations revealed that the PBD–ADN conjugate was poorly accommodated in the DNA minor groove of two DNA sequences containing the AGA-PBD binding motif, with the adenosine moiety of the ligand preventing the covalent binding of the PBD unit to the guanine amino group of the DNA duplex. These interesting findings shed further light on the ability of the substituents attached at the C8 position of PBDs to affect and modulate the biological and biophysical properties of PBD hybrids.

## 1. Introduction

**Figure 1:**
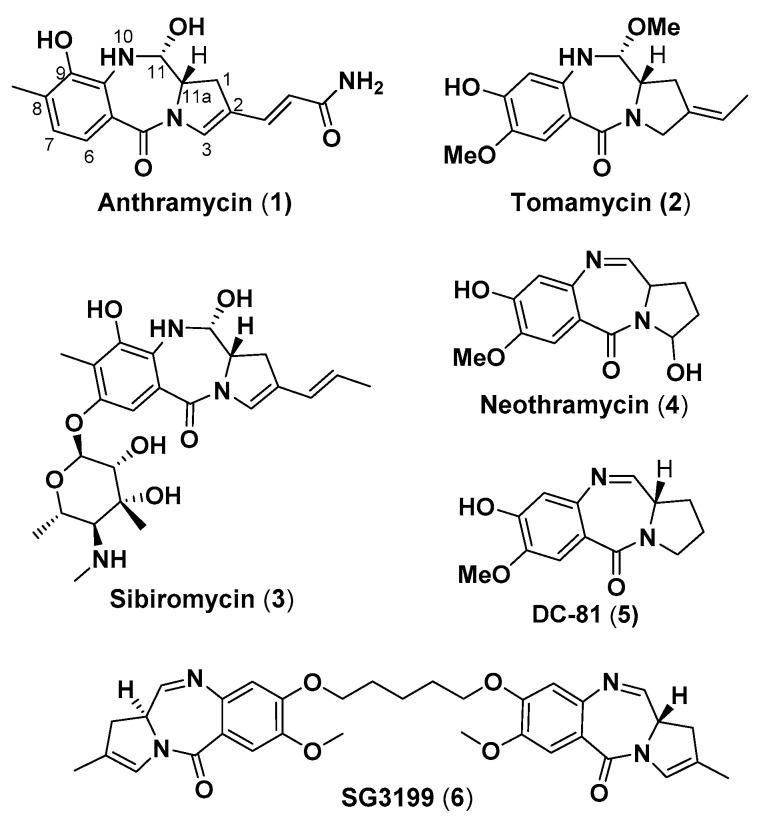
Structures of naturally occurring PBD monomers **1**–**5** and antibody–drug conjugate (ADC) warhead dimer **6**.

Pyrrolo(2,1-*c*)(1,4)benzodiazepines (PBDs) are naturally-occurring antitumor antibiotics produced by numerous actinomycetes species. Anthramycin (**1**) was the first PBD isolated from cultures of thermophilic actinomycete *Streptomyces refuineus* in 1965 [1], and since then several other PBDs have been discovered, i.e., tomaymycin (**2**), sibiromycin (**3**), neothramycin (**4**) and DC-81 (**5**) (Figure 1).

The mechanism of action of PBDs is associated with their ability to bind covalently to DNA and inhibit transcription factors and DNA replication activities [2,3,4]. PBDs interact with the walls and floor of the DNA-minor groove initially via a combination of hydrogen bonds, van der Waal forces and electrostatic interactions. This is followed by the formation of a covalent bond between the PBD imine or carbinolamine group (N10-C11) and the C2-NH_2_ of guanine residues resulting in a covalent amino linkage [5].

One of the main pharmacological and biological features of PBD lies within its C11a position, which is a chiral center with an *S*-configuration, imparting a right-handed twist to the molecule. This results in the desired three-dimensional shape allowing the PBDs to fit perfectly within the minor groove of DNA with minimal distortion in the DNA helix [6]. PBDs can selectively recognize and bind to DNA motifs containing “purine–guanine–purine” sequences [7,8], covalently binding to the central guanine base and forming hydrogen bonds, through the PBD-N10 hydrogen, with the adjacent purine [9]. Pyrrolobenzodiazepines can be divided in two subgroups, namely PBD monomers and dimers, the latter containing two PBD units tethered through alkyldioxy linkers. PBD dimer SG3199 [10] (**6**, Figure 1) is the warhead module of the antibody–drug conjugate (ADC) payload tesirine (SG3249) [11]. Rovalpituzumab-tesirine has recently completed phase I clinical trials for the treatment of small cell lung cancer [12].

The C8-position of the A ring of PBD is the most preferred point of attachment of substituents within the compound’s framework. Several chemical scaffolds, including heterocyclic polyamides [13,14,15], biaryl-units [16], benzofused rings [17] and quinazolinone [18] rings have been linked to the C8 position of PBD monomer producing compounds with improved DNA-sequence selectivity [13,16], and antimicrobial [19,20], anticancer [16] and antitubercular [21,22,23] activities compared to the PBD unit alone. The shape and physicochemical properties of C8-substituents have a direct effect on the cytotoxicity of the PBD-conjugates and their ability, or inability, to interact with the DNA-minor groove and inhibit transcription factors activity [13].

For example, the incorporation of heterocyclic polyamides to PBD rings via their C8 positions led to molecular hybrids with improved DNA-binding, antibacterial and anticancer properties [24]. In this instance, the polyamides, which possess intrinsic DNA sequence-selectivity and snugly fit within the DNA minor groove, act synergistically with the PBDs, leading to an overall improvement of the biological and biophysical properties of the two components of the hybrid [25].

On the other hand, if the pharmacophore structure linked to the PBD ring has a mechanism of action and a molecular target different from those of the alkylating agent, the resulting heterodimeric compounds is likely to exhibit a biological activity predominantly related to the C8-component of the conjugate. This is the case of PBD rings linked to aminopyrene [26], chalcone [27] or ciprofloxacin [23] motifs. A previously synthesized C8-linked PBD-aminopyrene conjugate was found to bind to G-quadruplex sequences, with the aminopyrene fragment driving the interactions with G-quadruplex DNA [28]. Indeed, the planar, electron-rich framework of pyrene allowed for ideal contacts with G-quadruplex structures resulting in the PBD ring portion of the conjugate not being able to bind to its DNA minor groove target [28].

Further to this, a recently reported C8-linked pyrrolobenzodiazepine–ciprofloxacin hybrid, which was found to inhibit the growth of *Mycobacterium tuberculosis* at micromolar concentrations, displayed neither DNA-binding activity nor eukaryotic cell toxicity [23]. Molecular modelling studies suggested the DNA gyrase enzyme, i.e., ciprofloxacin’s molecular target, to be the binding site of this PBD-conjugate [23]. It was apparent from this study that the DNA-alkylating properties of the PBD ring contained within the hybrid were completely ablated, whilst the antibiotic effects and mode of action of the compound were ascribable to the ciprofloxacin pharmacophore.

In this work, we wished to further investigate the contribution of the PBD unit to the PBD-conjugate’s biological activity, and an adenosine nucleoside was attached to the PBD A-ring C8 position through a pentynyl spacer. Adenosine serves a range of physiological roles in mammalian organisms and has been shown to mediate cytoprotection in response to cellular stress [29], whereas PBD pharmacophores have the opposite effect, causing cellular DNA damage and cell death. Incorporation of the cytoprotective nucleoside into a PBD-conjugate structure allowed us to gather additional evidence on the PBD moiety’s capability to modulate the biological activity of these hybrid molecules.

The novel adenosine-linked PBD hybrid was evaluated for DNA binding properties, cytotoxicity and anti-microbial activity. To our knowledge, this is the first example of a nucleobase linked to a PBD unit.

## 2. Chemical and Biological Experiments

### 2.1. Synthesis

**Scheme 1:**
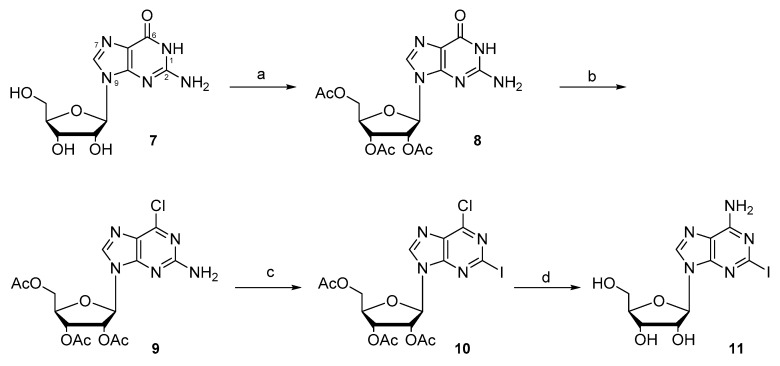
The synthesis of 2-iodoadenosine **11**. Reagents and conditions: (a) Ac_2_O, pyridine, DMF; (b) POCl_3_, Et_4_NCl, *N*,*N*-dimethylaniline, acetonitrile; (c) CuI, I_2_, CH_2_I_2_, isoamyl nitrite in THF, 80 °C; (d) NH_3_/MeOH.

The novel C8-linked PBD–adenosine conjugate **16** was prepared starting from 2-iodoadenosine (**11**). The latter was synthesized using a modified version of previously reported methods [30,31] from commercially available guanosine **7**, which was acetylated and then chlorinated to give the 6-chloro-guanosine triacetate **9** (Scheme 1). A Sandmeyer-type protocol involving a diazotization–iodination reaction was adopted to install the iodide group at the C2 position of the nucleoside, affording the aryl-iodide **10**. Amination of the 6-chloro-2-iodo-nucleoside **10** at its C6 position, and concurrent cleavage of the ribose sugar acetate groups, gave 2-iodo adenosine **11**.

**Scheme 2:**
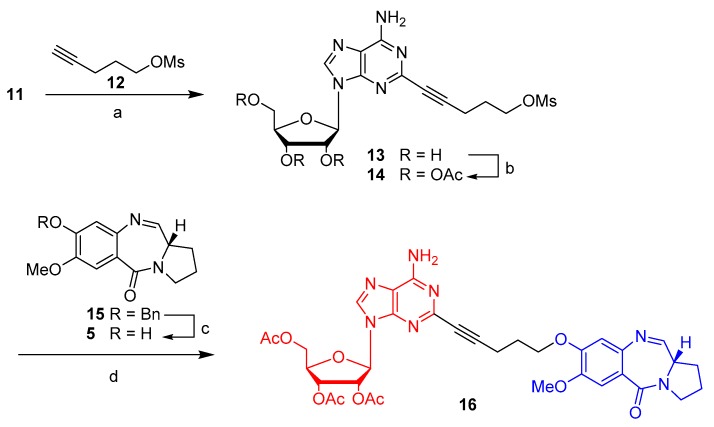
Synthetic approach for the preparation of **16**. Reagents and conditions: (a) pent-4-yn-1-yl methanesulfonate, (PPh_3_)_2_PdCl_2_, CuI, Et_3_N, ACN; (b) DMAP, Ac_2_O; (c) methanesulfonic acid, DCM, 0 °C to room temperature, 2 h; (d) **5**, KI, K_2_CO_3_, DMF.

The latter (**11**) was coupled with pent-4-yn-1-yl methanesulfonate **12** under Sonogashira cross-coupling reaction conditions to give **13** (Scheme 2). Subsequently, the three hydroxyl groups of the nucleobase were protected using DMAP and acetic anhydride to give adenosine triacetate **14**. Benzylated-PBD **15**, which was prepared in seven steps according to Tercel et al. [32] (please refer to the Supporting Information), was deprotected to provide DC-81 (**5**). PBD **5** was coupled with **14** to give final compound **16**.

### 2.2. Cytotoxicity Vs. Antimycobacterial Activity

The novel C8-linked PBD–adenosine hybrid (PBD–ADN) **16** and intermediates **5**, **11**, **15** were evaluated for cytotoxicity and growth inhibition activity against *Mycobacterium aurum* and *Mycobacterium bovis* BCG (Table 1), using the high throughput agar-based spot culture growth inhibition assay (HT-SPOTi) [33,34,35] that permitted a rapid determination of minimum inhibitory concentrations (MICs) of the compounds. The MIC_90_ values of the compounds tested against *M. aurum* and *M. bovis* BCG ranged from 500 to 3.91 mg/L. Isoniazid (INH) was used as a positive control and found to have MIC_90_ values of 7.81 mg/L and 0.49 mg/L for *M. aurum* and *M. bovis* BCG, respectively. The benzyl-protected pyrrolobenzodiazepine **15** displayed significant growth inhibition against both *M. aurum* and *M. bovis* with MIC_90_ values of 3.91 (11.6 µM) and 7.89 (23.4 µM) mg/L, respectively, whereas the PBD unit DC-81 (**5**), with a free C8- OH group, was moderately active against *M. bovis* with a MIC value of 31.25 mg/L. As expected, both PBDs **5** and **15** exhibited cytotoxicity against the murine macrophages. Interestingly, 2-iodoadenosine (2-I-ADN) **11** was found to be non-cytotoxic against the mammalian cells (GIC_50_ = 250 mg/L) and showed interesting anti-mycobacterial selectivity with a 10-fold higher activity against *M. bovis* (MIC = 15.63 mg/L, 39.7 µM) compared to *M. aurum* (MIC = 125 mg/L), thus confirming that purine analogues are promising scaffolds for the development of anti-microbial therapeutics [36,37]. PBD–ADN **16** displayed no cytotoxicity against RAW 264.7 cells and was found to be ineffective against the mycobacterial strains used in this screen. The compound’s selectivity index (SI) was calculated as the ratio between GIC_50_ and MIC_90_. The SI for INH and 2-I-ADN (**11**) for *M. bovis* were 1020 and 16, respectively, and **11** could be considered as a good anti-mycobacterial hit.

### 2.3. Footprinting Studies

**Figure 2:**
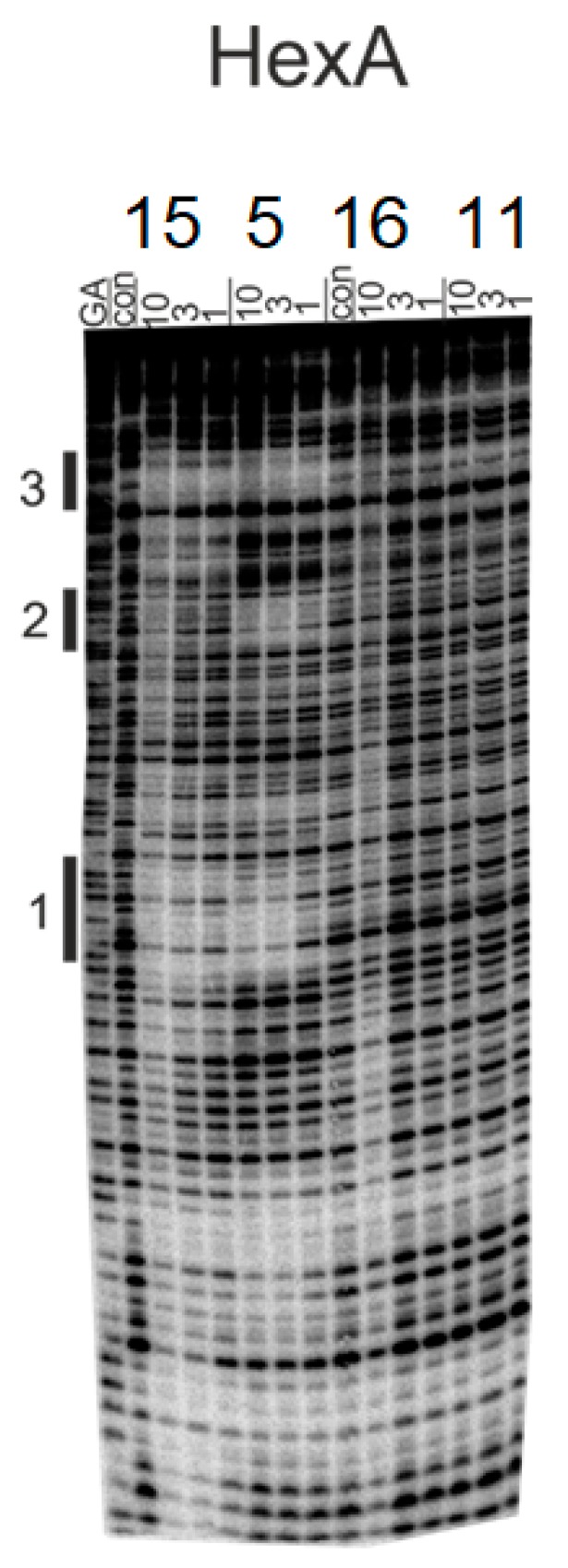
DNase I footprinting gels showing the interactions of **15**, **5**, **16** and **11** at 10, 3 and 1 µM concentrations with the HexA fragment. The samples were incubated with the ligand at room temperature for 24 hours before digesting with DNase I. Ligand concentrations are shown at the top of each gel lane. The tracks labelled “GA” correspond to a marker specific for purines, whereas “Con” indicates DNase I cleavage in the absence of the added ligands. Filled bars (with numbers) indicate the location of the footprints.

**Figure 3:**
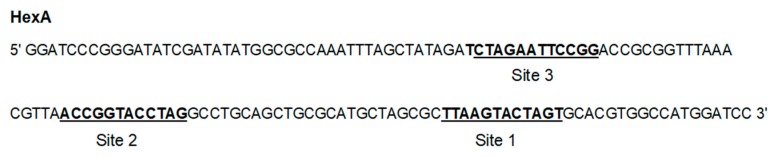
DNA sequences (underlined) of the footprinting substrates indicating the regions protected by the ligand from DNase I cleavage.

The DNA sequence selectivity of the novel PBD–adenosine conjugate **16** and its intermediates **11** (2-I-ADN), **5** (DC-81) and the benzylated-PBD **15** were investigated at concentrations of 10, 3 and 1 µM using DNA fragment HexA, which contain several symmetrical hexanucleotide sequences [38]. The DNase I footprints for the interactions of **16**, **11**, **5** and **15** with HexA are illustrated in Figure 2 and the DNA sequences protected from cleavage by the ligands are shown in Figure 3.

In HexA, only PBDs **5** and **15** produced clear footprints at 5′-TTAAGT/ACTAGT (site 1), 5′-ACCGGT/ACCTAG (site 2) and 5′-CTAGAA/TTCCGG (site 3). DC-81 (**5**) bound to site 1 at 3 µM and to sites 2 and 3 at the lowest concentration (1 µM), whereas benzylated PBD **15** only gave weak protection from DNase I cleavage at sites 1 and 3 at 10 and 3 µM. Examination of the footprints with the DNA fragments revealed the presence of the preferred PBD binding motif, Pu–G–Pu, for ligands **5** and **15** in site 3 of HexA (5′-AGA and 5′-GGA) and all sites containing 5′-AG (5′-CT). Notably, the novel PBD adenosine conjugate did not produce any footprints.

### 2.4. Molecular Modelling

**Figure 4:**
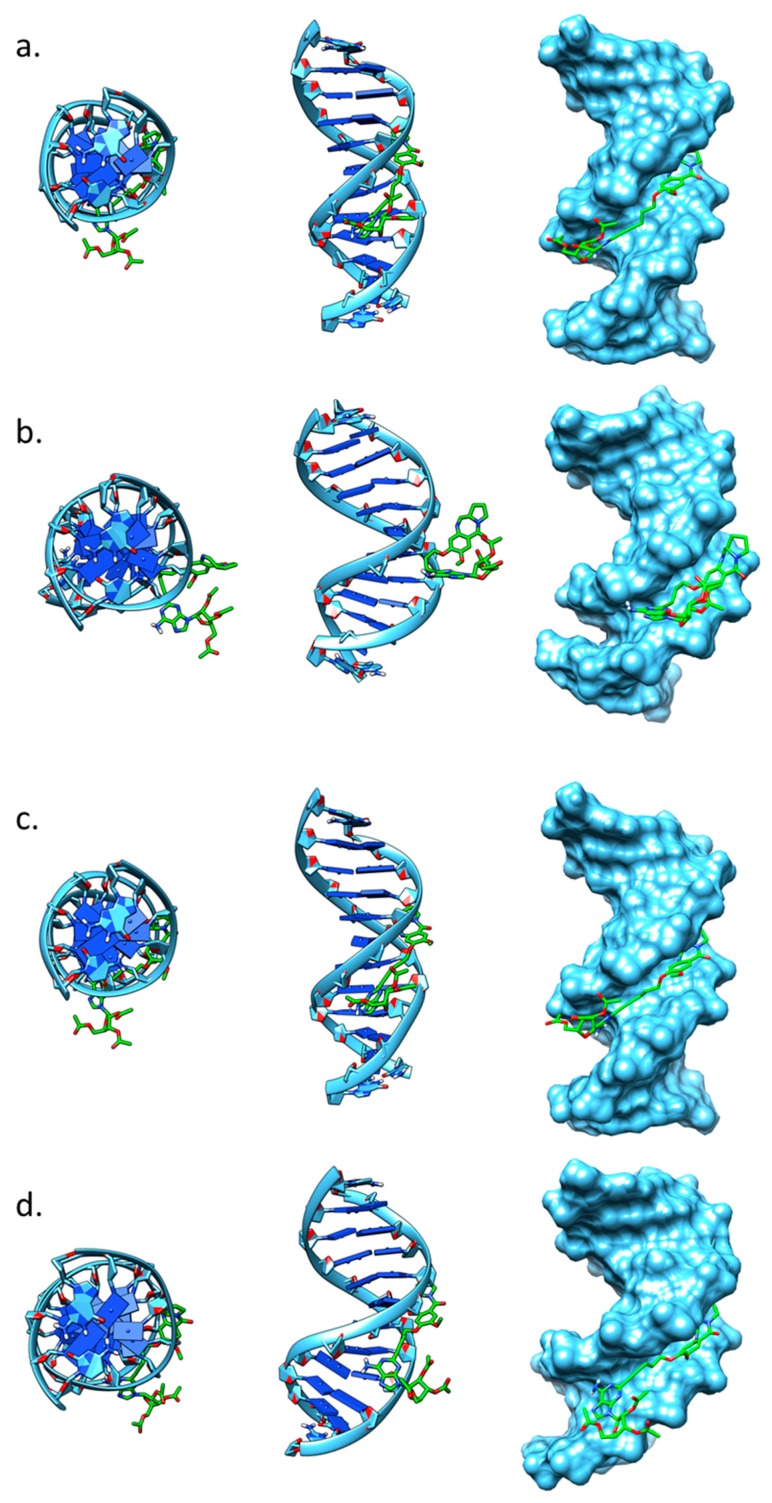
Images from molecular dynamics simulations of compound **16** bound to 12mer DNA sequences. Top down (left), side (center) and surface (right) representations of the DNA with the compound **16** (green). (**a** and **b)**. DNA sequence 5′-CGTAGATTTGCG-3′ before (**a**) and after (**b**) 30 ns of solvated molecular dynamics; (**c** and **d)** DNA sequence 5′-CGTAGATCTGCG-3′ before (**c**) and after (**d**) 30 ns of solvated molecular dynamics.

In an attempt to explain the non-DNA-binding behaviour of PBD hybrid **16**, molecular modelling studies were conducted. Two solvated molecular dynamics simulations were prepared in which compound **16** was positioned in the minor groove of two DNA sequences (5′-CGTAGATTTGCG-3′ and 5′-CGTAGATCTGCG-3′) to mimic the pre-covalent complex formed between the PBD and the guanine residue of an AGA-PBD binding motif. Both simulations suggested that the ligand was poorly accommodated in the minor groove (Figure 4). The initial complex prepared by positioning compound **16** in the minor groove adjacent to the exocyclic amine of guanine suggested that the linker at the 8-position of the PBD would project along the minor groove of the DNA towards the 3′ end, with the purine positioned close to the TTT or TCT sequences of the respective DNA sequences and the acetylated ribose pointing out of the minor groove towards the solvent (Figure 4a,c). After a total of 30 ns of solvated molecular dynamics (5 ns equilibration and 25 ns production) the complexes had either shifted within the minor groove or partially detached (Figure 4b,d). The simulations suggest that the bulkiness of the adenine and the linker geometry may be responsible for the relatively poor binding to the minor groove. Further studies would be required to confirm this.

## 3. Conclusions

C8-linked PBD-conjugates are an important class of anti-tumor and antibiotic compounds. Several examples of this type of molecules have been reported in the literature in the past decades, although it is unclear to what extent the PBD pharmacophore contributes to the overall biological activity of the heterodimers. Here, we sought to explore the role of PBD units in the biological activity of PBD-hybrids, and a cytoprotective molecule, adenosine, was appended to a PBD ring. The novel C8-linked PBD–adenosine (PBD–ADN) hybrid (**16**) was prepared using a convergent synthetic approach and screened for antimicrobial, antiproliferative and DNA-binding activities. The adenosine (**11**) and the PBD DC-81 (**5**) units were synthesized separately and then linked via a pentynyl spacer at a later stage to give the title compound **16**. The new ligand displayed no cytotoxicity against mammalian cells and was not active against *M. aurum* and *M. bovis* strains. The inclusion of an acetylated purine nucleoside to the PBD framework might be expected to either favor the interactions with the DNA minor groove’s walls and floor or allow for contacts in the major groove in a triple-helix-forming-oligonucleotides fashion. However, the PBD–ADN conjugate did not bind, even at high concentrations, to any DNA sequences in the HexA fragment. This interesting finding, which will have an impact on future PBD conjugates design, gives further insights on the ability of the C8-linked substituents to modulate the DNA binding properties of the hybrid compound. Molecular dynamics simulations showed that the adenosine moiety was not favorably accommodated within the minor groove of a 12-mer DNA duplex, thus preventing the PBD unit from binding to its AGA binding motif. It is anticipated that the PBD–ADN hybrid might not be able to reach its target sites (i.e., DNA) in either eukaryotic or bacterial cells, with the adenosine moiety preventing the PBD-conjugate from binding to naked DNA structures. Additional studies, including the deprotection of the acetate groups of the adenosine ribose moiety and the introduction of more flexible or less hydrophobic linkers, are underway to prepare analogues to further investigate the PBD–ADN conjugate biological and biophysical activities.

## 4. Materials and Methods

### 4.1. General Chemistry Reactions Information

Chemicals were purchased from Acros Organic, Alfa Aesar, Fisher Scientific, Sigma Aldrich and VWR. The deuterated solvents (CDCl_3_, DMSO-*d*_6_ and MeOD-*d*_4_) used for NMR spectroscopy experiments were purchased from Cambridge Isotope Laboratories Inc. Nitrogen gas was purchased from BOC and used as received. Synthetic reactions, involving moisture or air sensitive reagents, were performed under nitrogen atmosphere using standard Schlenk line techniques. For moisture-free reactions, glassware was pre-dried in an oven at 100 °C overnight and flame-dried under vacuum immediately before use. Melting points (m.p.) were recorded on a SMP20 Cole-Palmer digital melting point apparatus and were uncorrected. LC–MS analysis was conducted on a Thermo Fisher Agilent 6100 series Quadrupole LC–MS system equipped with a G4220A 1290 binary pump/DAD, using an Agilent Zorbax SB-C19 2.1 × 50 mm, 1.8-µm, 600 bar HPLC column. Flow: 1 mL/min. Solution A: H_2_O 0.1% formic acid; Solution B: acetonitrile (ACN) 0.1% formic acid. High pressure typically starting at about 500 psi (13.60 min run): solution B was kept at 5% for 2 min and increased to 100% up to 9 min. This concentration (100% ACN) was held for 2 min, then reduced to 5% over 0.5 min and kept at 5% until the end of the run. Nuclear magnetic resonance (NMR) spectroscopy analyses of the synthetic compounds were carried out using a Bruker AV3 400 MHz instrument with a 9.4 T Ultra Shield magnet. Solvent signals for hydrogen and carbon NMR were used as the internal reference. Chemical shifts *(δ*_H_) are quoted in parts per million. Coupling constants (*J*) are given in Hertz (Hz) and the signal multiplicity is described as singlet (s), broad singlet (brs), doublet (d), doublet of doublets (dd), triplet (t), doublet of triplets (dt), quartet (q), doublet of quartets (dq) and multiplet (m).

Please refer to the Supporting Information for the detailed synthetic procedures of **5**, **13** and **14**.

*(2R,3R,4R,5R)-2-(Acetoxymethyl)-5-(6-amino-2-(5-(((S)-7-methoxy-5-oxo-2,3,5,11a-tetrahydro-1H-benzo(e)pyrrolo(1,2-a)(1,4)diazepin-8-yl)oxy)pent-1-yn-1-yl)-9H-purin-9-yl)tetrahydrofuran-3,4-diyl diacetate* (**16**). A solution of **5** (12 mg, 0.048 mmol) in dimethylformamide (0.5 mL) was added to a mixture of **14** (14 mg, 0.025 mmol), potassium carbonate (20 mg, 0.14 mmol) and potassium iodide (8 mg, 0.048 mmol). The reaction mixture was stirred for 36 h at 30 °C and monitored by LC–MS analysis. Solid residues were filtered off, washed with dimethylformamide (0.2 mL) and then discarded. The reaction mixture was concentrated to dryness and purified by semi-preparative reverse-phase HPLC using a Phenomenex Gemini 5 µm C18 110 Å (250 × 10.0 mm) column (Phenomenex). Mobile phase A was UHP water, while mobile phase B was acetonitrile. Conditions: 60% A from 0 to 0.5 min, after which the percent B was increased to 100% over 20 min. Subsequently, B was kept at 100% for 10 min providing a total run time of 30 min. The flow rate was 1 mL/min and the injection volume was 50 µL. The appropriate fractions were collected and freeze dried to give the title compound **16** (3.5 mg, 34%) as an off-white solid. ^1^H-NMR (400 MHz, CD_3_OD): δ_H_ 8.24 (s, 1H, adenine H-8), 7.57 (s, 1H, PBD-H-9), 7.13 (s, 1H, PBD-H-6), 6.18 (d, 1H, J = 5.1 Hz, ribose H-1′), 5.92 (t, 1H, J = 5.3 Hz, ribose H-4′), 5.72 (t, 1H, J = 5.1 Hz, ribose H-3′), 4.43-4.39 (m, 2H, ribose H-2′ and H-5′), 4.37-4.33 (m, 2H, linker CH_2_O), 3.94 (s, 3H, OCH3), 3.79 (d, J = 1.5 Hz, 1H, PBD H-3), 3.65-3.63 (m, 1H, PBD H-3), 3.14 (t, J = 8.1 Hz, 1H), 2.72 (t, J = 7.1 Hz, 1H, linker CH_2_), 2.33- 2.25 (m, 2H, linker CH_2_), 2.23-2.17 (m, 2H, PBD H-2), 2.12 (s, 3H, OAc), 2.05 (s, 3H, OAc), 2.03 (s, 3H, OAc), 0.92-0.87 (m, 2H, PBD H-1). ^13^C-NMR (100 MHz, CD_3_OD): δ_C_ 172.2, 171.4, 171.2 (C=O), 162.1 (PBD C-5), 160.5 (PBD C-11), 157.1 (adenosine C-6), 154.1 (adenosine C-2), 141.4, 116,2, 110.6 (PBD C-9), 108.7 (PBD C-6), 103.1, 97.0, 87.8 (ribose C-1′), 81.7 (ribose C-2′), 74.5, 71.9 (alkyne C-C), 68.1, 64.1 (CH_2_OAc), 60,4, 56.9 (OCH_3_), 50.2 (PBD C-3), 31.6 (CH_2_ linker), 30.3 (PBD C-1), 23.8 (PBD C-2), 20.7 (-OAc CH_3_), 20.4 (-OAc CH_3_), 20.3 (-OAc CH_3_), 16.4 (alkyne CH_2_); MS m/z 704 (M+); HRMS calc. for C_34_H_37_N_7_O_10_ 703.2602, found 704.2621 (M·+ H^+^).

### 4.2. DNAse I Footprinting Assay

Footprinting reactions were performed as previously described [38] using the DNA fragments HexAfor. The DNA fragments were obtained by cutting the parent plasmids with *Hind*III and *Sac*I (*Hex*A) or *Eco*RI and *Pst*I (*Hex*BRev) and were labelled at the 3′-end with (α-^32^P)dATP using reverse transcriptase. After gel purification the radiolabeled DNA was dissolved in 10 mM Tris-HCl pH 7.5 containing 0.1 mM EDTA, at a concentration of about 10 c.p.s per µL as determined on a handheld Geiger counter. An aliquot of 1.5 µL of radiolabeled DNA was mixed with 1.5 µL ligand that had been freshly diluted in 10 mM Tris-HCl pH 7.5, containing 10 mM NaCl. The complexes were left to equilibrate for at least 12 h before digesting with 2 µL DNase I (final concentration about 0.01 units/mL). The reactions were stopped after 1 min by adding 4 µL of formamide containing 10 mM EDTA and bromophenol blue (0.1% *w*/*v*). The samples were then heated at 100 °C for 3 min before loading onto 8% denaturing polyacrylamide gels containing 8 M urea. Gels were fixed in 10% acetic acid, transferred to 3MM paper, dried and exposed to a phosphor screen overnight, before analyzing with a typhoon phosporimager.

### 4.3. Microbiology and Cytotoxicity Experiments

#### 4.3.1. Mycobacteria

Slow growing *Mycobacterium bovis* BCG (ATCC35734) and relatively fast growing *Mycobacterium aurum* (ATCC 23366) were used for anti-mycobacterial activity of the chemical compounds studied in this work.

#### 4.3.2. Preparation of Microbiological Media

All the sterile microbiological prepared solid and liquid media was stored in a dark cabinet for a maximum time of 1 month and two weeks for Middlebrook 7H9 broth. After the expiration time had elapsed, the media was discarded. The sterility of the broth observed by checking the transparency of the liquid.

Middlebrook 7H9. An accurate weight of 0.94 g of Middlebrook 7H9 was added to 180 mL of deionized water (MilliQ) and dissolved completely by shaking. Subsequently, 800 µL of 50% glycerol solution in deionized water was added to the media to make a final concentration of 0.05% (*v/v*). The preparation was autoclaved at 121 °C (15 psi) for 12 min. Finally, 10% (*v/v*) Middlebrook ADC enrichment was added to the medium just before inoculation.

Middlebrook 7H10. An accurate weight of 3.8 g of Middlebrook 7H10 was added to 180 mL of deionized water (MilliQ) and dissolved completely by shaking vigorously. Subsequently, 2 mL of 50% glycerol solution in deionized water was added to the media to make a final concentration of 0.5% (*v/v*). The preparation was autoclaved at 121 °C (15 psi) for 12 min. Finally, 10% (*v/v*) Middlebrook OADC enrichment was added to the medium just before inoculation.

#### 4.3.3. Growth, Maintenance and Cryopreservation of *M. aurum*

The incubation of cultures of mycobacterium species was performed in a Sartorius Certomat 15 shaking incubator. For culture of RAW 264.7 macrophage cell line, a CO_2_ Excella ECO170 incubator was used. Ultracentrifugation was carried out in a Beckman Coulter J-26XP Avanti centrifuge with temperature control. Low speed centrifuge was performed in ThemoScientific Heraeus Megafuge 16R.

*M. aurum* was grown in Middlebrook 7H9 broth with 0.2% glycerol (*v/v*), 0.05% Tween-80 (*v/v*) and supplemented with 10% ADC (*v/v*). From a cryopreserved stock, a 1:1000 dilution in fresh media was performed. The volume of liquid media should be one fifth of the total volume of the container to allow space for cell respiration. *M. aurum* was grown at 37 °C in an incubator shaker at 180 rpm. Colonies on Middlebrook 7H10 agar media were observed after five days.

Cultures of bacterial organisms were preserved by suspending 500 µL of culture in 500 µL of sterile 50% *v/v* glycerol aqueous solution. The mixture was dispensed into 2 mL cryovials (Nalgene), which were closed with a rolling lid. The vials were mixed by inverting, allowing distribution of the cells in the fluid. The cryovials were stored in a −80 °C freezer.

#### 4.3.4. Optical Density of Bacterial Culture

The optical density (OD_600_) was measured in a spectrophotometer at 600 nm by dispensing 1 mL of sterile media in a plastic cuvette and used as a reference to blank the spectrophotometer. An aliquot of 1 mL of the liquid culture was dispensed into a plastic cuvette and the reading was taken. Readings were taken every 12 h to obtain a growth curve.

##### Growth Curve

*M. aurum* was grown until mid-log phase in the 50 mL falcon tube containing media supplemented with ADC, at 37 °C, 180 rpm. Two 250 mL flasks were autoclaved. The flasks contained 45 mL of M7H9 to which 5 mL of ADC was supplemented alongside 500 μL of culture. The optical density was taken every 12 h and plotted in a graph with time in the *x*-axis and optical density at 600 nm in the *y*-axis.

#### 4.3.5. MIC Determination Using Solid Agar Method (Spot Culture Growth Inhibition Assay)

##### Preparation of the Master Plate

To a PCR tube plate, 10 µL of DMSO was added to each well. The samples were weighed ensuring a final concentration of 50 mg/mL. The samples were dissolved in DMSO and 10 µL of the drug was added to the first well. Subsequently, 10 µL of solution was removed from the first well and added to second row and serially diluted accordingly. From the master plate, 2 µL was taken and added to the SPOTi plate. The master plate was sealed and kept in −20 °C freezer.

##### Preparation of Spot Culture Growth Inhibition Assay

Log-phase cultures of *M. aurum* or *M. bovis BCG* were grown until OD_600_ reached 0.8–1.0 and diluted to 10^−3^ in M7H9 media. Middlebrook 7H10 supplemented with oleic acid–albumin–dextrose–catalase supplement (OADC) was added. Subsequently, 200 µL of the molten agar was added to the 96 well plate by using MultidropTM Combi Reagent Dispenser (Thermo Scientific, UK) and shaken to mix the drug with the agar. The plates were left to settle. At this stage, the diluted cells were added through the plate dispenser (2 µL in each well). The cell growth at 37 °C was recorded after 5 days for *M. aurum* and 14 days for *M. bovis* BCG. Wells with DMSO were used as negative controls and a row with isoniazid was used a positive control.

#### 4.3.6. Resazurin Cytotoxicity Assay

The resazurin assay relies on colorimetric cell viability. In its oxidized (blue) form, the dye enters the cell cytosol where it is reduced to resorufin resulting in a color change (red). The blue oxidized form relates to cell death whereas the red/pink reduced form indicates cell proliferation. Raw 264.7 macrophage cells were grown in RPMI-1640 medium enriched with 2 mM L-glutamine and 10% heat inactivated fetal bovine serum (*v/v*) and passaged twice before the assay. The assay was performed in 96-well cell culture flat bottom plates in triplicate.

Firstly, 2 µL of the 50 mg/mL final stock solution of compounds was added to 200 µL of complete RPMI-1640 medium in the first row and then a two-fold serial dilution was carried out. An aliquot of 100 µL of diluted macrophage cells (5 × 10^5^ cells/mL) was added to each well and incubated at 37 °C for 48 h maintaining 5% CO_2_. The cells were washed twice with fresh PBS and fresh complete RPMI-1640 medium was added. A solution of 0.01% resazurin solution was freshly prepared, and 30 µL of the resazurin solution was added to the wells and incubated overnight at 37 °C (in CO_2_ incubator). The following day the change in color was observed, and the fluorescence intensity was measured at λ_exc_560/λ_emi_590 nm using a FLUOstar OPTIMA micro plate reader. The 50% growth inhibitory concentration (GIC_50_) was determined based on a resazurin fluorescence assay and the selectivity index (SI), was determined as the ratio between the GIC_50_ on RAW 264.7 macrophages and the MIC on *Mycobacterium* spp.

### 4.4. Molecular Dynamics Methods

Two B-form 12mer DNA duplexes (5′-CGTAGATTTGCG-3′ and 5′-CGTAGATCTGCG-3′), which incorporate one or two PDB binding sites, respectively (AGA or TCT on the opposing strand), were constructed using NAB in AmberTools [39]. Compound **16** was constructed with appropriate stereochemistry in ChemDraw and then converted to a 3D format using Chem3D and optimized using the default MM2 and MMFF94 energy minimization protocols. The compounds were saved as .pdb files and parameterized (atom types, partial charges) using antechamber from AmberTools and saved as .prep Amber input files. The ligands were manually positioned in the minor groove of the DNA duplex so that the imine C11 was adjacent to the exocyclic amine of the guanine residue of the PBD binding site. Linker positions and dihedral angles in the ligands were edited manually to remove clashes with the minor groove and to optimize interactions as appropriate. Distance restraints were applied to the PBD so that the imine was held within 2.5 Å of the adjacent guanine and adenine residues. The structure was energy minimized (steepest descent and conjugate gradient) using Sander (Amber 18). The structure was then equilibrated to 300 K with a constant 0.5 kJ/mol energy restraint applied to the DNA. The structures were subject to 2 ns of GBSA molecular dynamics with the ligand–DNA and DNA restraints in place, then a further 2 ns of GBSA molecular dynamics with DNA restraints, but no ligand–DNA restraints. The final structures were converted to .pdb files and reparametrized for MD using tleap with the inclusion of a 10 Å TIP3P water box with Na^+^ ions to neutralize the system. The complexes were run through a standard four stage minimization protocol then heated to 300 K and the restraints on the DNA relaxed over 8 sequential stages using pmemd from Amber18. Unrestrained molecular dynamics were run for 5 ns, and then trajectories were collected over a further 25 ns of simulation time. The trajectories were centered, imaged and stripped of the counterions and solvent using cpptraj (AmberTools). The interaction energies were calculated using MMPBSA (GBSA method) and the trajectories were observed in VMD [40] to note any structural changes or dissociation of the complexes. Images were prepared using UCSF Chimera [41].

## Figures and Tables

**Table 1:** Anti-mycobacterial and cytotoxic activity evaluation of **5**, **11**, **15** and **16** using the high-throughput spot-culture growth inhibition assay (HT-SPOTi) technique.

Compound	GIC_50_^a^ RAW 264.7 (mg/L)	MIC_90_^b^ *M. aurum* (mg/L)	MIC_90_ *M. bovis* BCG (mg/L)	SI ^c^ GIC_50_/MIC_90_ *M. bovis*
PBD (**5**)	0.49	62.5	31.25	0.01
Bn-PBD (**15**)	0.48	3.91	7.89	0.06
2-I-ADN (**11**)	250	125	15.63	16
PBD–ADN (**16**)	500	500	250	2
INH	500	7.81	0.49	1020

^a^ GIC_50_ is the concentration of the compounds at which 50% of maximal inhibition of cell proliferation is achieved using resazurin-based micro dilution method on murine macrophages RAW 264.7. ^b^ MIC_90_ is the lowest concentration of the compound at which 90% of the bacteria was inhibited. ^c^ SI is the ratio between GIC_50_ and the MIC_90 *M. bovis*_.

## Supplementary Materials

The Supplementary Materials are available online.
