# Supplementary material for: Synthesis and Biological Evaluation of a Novel C8-Pyrrolobenzodiazepine (PBD) Adenosine Conjugate. A Study on the Role of the PBD Ring in the Biological Activity of PBD-Conjugates
Source: Molecules. 2020 Mar 10;25(5):1243. doi: 10.3390/molecules25051243 (PMC7179398; doi:10.3390/molecules25051243)
Supplement: Supplementary file 1 [file molecules-25-01243-s001.pdf]

Supporting information

Synthesis and biological evaluation of a novel C8-pyrrolobenzodiazepine(PBD) adenosine conjugate. A study on the role of the PBD ring in the biological activity of PBD-conjugates.

Lindsay Ferguson, Sanjib Bhakta, Keith R. Fox, G. Wells and Federico Brucoli

Table of contents

Scheme 1. Synthesis of PBD monomer DC-81 (**5**)……………………………..Page S2

Scheme 2. Synthesis of 2-iodoadenosine (**11**)………………………………...Page S12

Appendix I. Spectral data for **16**………………………………………………Page S19

Synthesis of PBD monomer DC-81 (**5**)

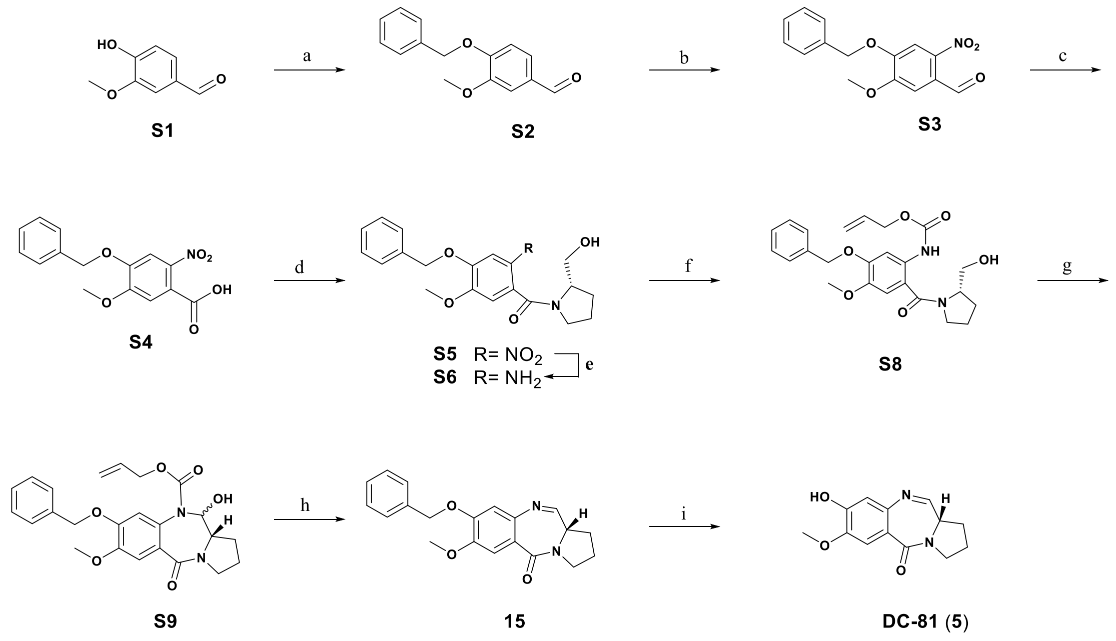

Scheme S1. *Reagents and conditions:* (a) BnCl, K_2CO_3, EtOH; (b) HNO_3, $(CH_3CO)_2O$; (c) $NaClO_2$, H_2O_2, H_2O/ACN; (d) $(COCl)_2$, DMF/DCM, (*S*)-2-pyrrolidinemethanol, DCM; (e) NH_2-NH_2, Raney-Ni, CH_3OH; (f) allyl chloroformate, pyridine, DCM; (g) TEMPO, DAIB, DCM; (h) $Pd(PPh_3)_4$, pyrrolidine, DCM; (i) methanesulfonic acid.

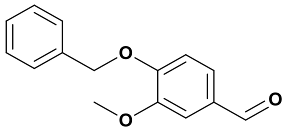

4-benzyloxy-3-methoxybenzaldehyde (S2).

To a well stirred solution of vanillin (20 g, 131 mmol) in absolute ethanol (115 mL), K_2CO_3 (20 g, 144 mmol, 1.1 equiv.) and benzyl chloride (16.6 mL, 131 mmol, 1 equiv.) were added drop wise at 0 °C. The reaction was heated to reflux and maintained under nitrogen atmosphere overnight. The reaction was quenched with 30 mL of distilled water, which was added dropwise. The mixture was dissolved in EtOAc (250 mL), filtered and washed with 10% NaOH (3 × 75 mL) and brine (3 × 90 mL) and dried using $MgSO_4$. The solvent was evaporated under reduced pressure to produce pure **S2** (28.85 g, 91%). R_f = 0.4 (Hexane:EtOAc / 7:3 *v/v*); MS *m/z* 242.1 (M^+); ^{1}H-NMR (400 MHz, $CDCl_3$) δ_H 9.84 (s, 1H, CHO). 7.46-7.31 (m, 7H, Ar-H), 6.99 (d, *J* = 8.09 Hz, Ar-H), 5.25 (s, 2H, $ArCH_2O$), 3.95 (s, 3H, $-OCH_3$); ^{13}C-NMR (100 MHz, $CDCl_3$) δ_C 190.8 (C=O), 128.9 (C=C), 128.2 (C=C), 127.2 (C=C), 126.5 (C=C), 112.4 (C=C), 109.4 (C=C), 70.9 (CH_2), 56.0 (CH_3).

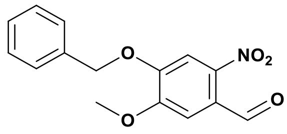

4-(benzyloxy)-5-methoxy-2-nitrobenzaldehyde (S3).

A solution of 4-benzyloxy-3-methoxybenzaldehyde **S2** (28 g, 0.118 mol) in acetic anhydride (100 mL) was added dropwise over a 30-minute period to a solution of 70% nitric acid (445 mL) in acetic anhydride (100 mL) at 0 °C. The mixture was stirred for 3.5 hours at 0 °C. Then, the reaction mixture was poured into ice-water (2 L) to produce a yellow precipitate which was filtered and washed with distilled water. The yellow solid was then dried in an oven overnight at 40 °C to give pure **S3** as a yellow powder (22.4 g, 66%); R_f = 0.56 (Hexane:EtOAc / 7:3 *v/v*); MS *m/z* 287.6 (M^+); ^{1}H-NMR (400 MHz, $CDCl_3$) δ_H 10.43 (s, 1H, CHO), 7.67 (s, 1H, H-3), 7.47-7.33 (m, 6H, Ar-H), 5.27 (s, 2H, $ArCH_2O$), 4.02 (s, 3H, OCH_3); ^{13}C-NMR (100 MHz, $CDCl_3$) δ_C 187.7 (C=O), 128.9 (C=C), 128.7 (C=C), 127.6 (C=C), 110.0 (C=C), 108.9 (C=C), 71.6 (CH_2), 56.7 (CH_3).

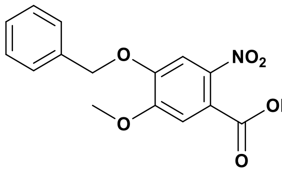

4-Benzyloxy-5-Methoxy-2-Nitro-Benzoic Acid (S4).

Pinnick oxidation method

A solution of $NaClO_2$ (8 g, 87 mmol, 5 equiv.) in H_2O (75 mL) was slowly added at 0 °C to a solution of 4-(benzyloxy)-5-methoxy-2-nitrobenzaldehyde **S3** (5 g, 17.4 mmol), NaH_2PO_4 (5 g, 34.8 mmol, 2.5 equiv.) and H_2O_2 (4.65 mL) in acetonitrile:H_2O 3:2 (100 mL). The reaction mixture was stirred at room temperature for 6 h until no more oxygen evolved from the reaction. After acidification with 10% HCl to pH = 3, the solution was extracted with EtOAc (3 × 30 mL), dried over $MgSO_4$ and evaporated under reduced pressure to yield **S4** (5.6 g, 66%) of product. R_f = 0.25 (Hexane:EtOAc:Acetic acid / 6:3:1 *v/v*); MS *m/z* 303.5 (M^+); ^{1}H-NMR (400 MHz, DMSO-δ_6) δ_H 13.64 (brs, 1H, COOH), 7.69 (s, 1H, H-3), 7.47-7.36 (m, 6H), 7.31 (s, 1H. H-6), 5.24 (s, 2H, CH_2), 3.92 (s, 3H, OCH_3). ^{13}C-NMR (100 MHz, DMSO) δ_C 165.9 (COOH), 152.0 (C=C), 148.2 (C=C), 141.1 (C=C), 135.8 (C=C), 128.5 (C=C), 128.2 (C=C), 128.0 (C=C), 127.9 (C=C), 121.5 (C=C), 111.4 (C-3), 108.5 (C-9), 70.5 ($ArCH_2O$), 56.4 (OCH_3).

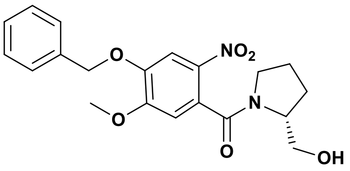

(4-(benzyloxy)-5-methoxy-2-nitrophenyl)(2-(hydroxymethyl)pyrrolidin-1-yl)methanone (S5).

Oxalyl Chloride method

The 4-benzyloxy-5-methoxy-2-nitro-benzoic acid **S4** (5 g, 16.5 mmol) was dissolved in 30 mL of anhydrous acetonitrile. To this solution, oxalyl chloride (2.4 mL, 18.15 mmol, 1.1 equiv.) and a catalytic amount of DMF were added. The reaction was stirred at room temperature overnight to prepare the acid chloride derivative. The acid chloride solution was added dropwise over one hour to a solution of *S*-pyrrolidinemethanol (2 mL, 20 mmol, 1.2 equiv.) and TEA (3.65 mL, 36.47 mmol, 2.21 equiv.) in anhydrous acetonitrile under a nitrogen atmosphere at a temperature of -30 ^{o}C throughout using a liquid nitrogen and chloroform slush. The reaction was allowed to reach room temperature and stir overnight. The solution was then extracted with $CHCl_3$ (4 × 100 mL) and the combined organic fractions were washed with 1 M HCl (2 × 50 mL), H_2O (2 × 75 mL), brine (2 × 50 mL) and H_2O (2 × 50 mL), dried using $MgSO_4$ and the solvent evaporated *in vacuo* to afford a yellow oil. The oil was purified by flash chromatography ($CHCl_3$ then gradient to 5% MeOH-$CHCl_3$) to afford **S5** (3.19 g, 50%) as a pale-yellow oil, which slowly crystallised on standing. R_f = 0.45 (DCM:MeOH / 9.5:0.5 *v/v*); $[\alpha]^{20}_{D}$ = +62.2 (*c* = 0.45, $CHCl_3$); MS m/z 387.3 (M^+); ^{1}H-NMR (400 MHz, $CDCl_3$) δ_H 7.77 (s, 1H, H-9), 7.45-7.23 (m, 5H, Ar-H) , 6.83 (s, 1H, H-6), 5.23 (s, 2H, benzyl-CH_2), 4.39 (bs, 1H, OH), 3.92 (s, 3H, OCH_3), 3.88-3.80 (m, 2H), 3.19-3.16 (t, 2H, H-3), 2.20-1.26 (m, 4H, H2-H4), 1.31-1.11 (m, 1H).

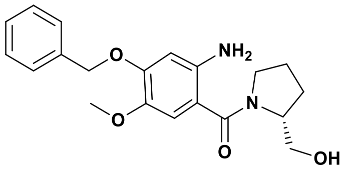

(2-amino-4-(benzyloxy)-5-methoxyphenyl)(2-(hydroxymethyl)pyrrolidin-1-yl)methanone (S6).

Hydrazine hydrate (1 mL, 31 mmol, 5 equiv.) was added dropwise to a solution of **S5** (2.4 g, 6.2 mmol) in dry methanol (10 mL) and a catalytic quantity of Raney Nickel (240 mg) over anti-bumping granules. A gentle reflux was maintained for 30 mins when TLC (5% MeOH-$CHCl_3$) indicated that the reaction had gone to completion. The Ni catalyst was then removed by filtration through Celite and the solvent concentrated *in vacuo*. Purification by flash chromatography (5% MeOH-$CHCl_3$) afforded the amine **S6** as a bright yellow unstable oil, which required storage at low temperature (1.83 g, 83%). R_f = 0.43 (EtOAc); MS *m/z* 357.1 (M^+); ^{1}H-NMR (400 MHz, $CDCl_3$) δ_H 7.42 - 7.30 (m, 5H, Ar-H), 6.76 (s, 1H, H-6), 6.26 (s, 1H, H-9), 5.11 (s, 2H, $ArCH_2O$), 4.39 (bs, 1H, OH), 3.80 (s, 3H, OCH_3), 3.70-3.59 (m, 2H), 3.54-3.46 (m, 2H), 2.91 (d, *J* = 26.8 Hz, 1H, H-2), 2.18-2.11 (m, 1H,), 1.91-1.83 (m, 1H), 1.78-1.61 (m, 3H).

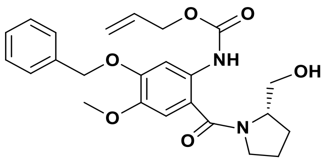

(*S*)-allyl-(5-(benzyloxy)-2-(2-(hydroxymethyl)pyrrolidine-1-carbonyl)-4-methoxyphenyl)carbamate (S7).

Allyl chloroformate (0.62 mL, 5.2 mmol, 1.05 equiv.) was added dropwise to a solution of pyridine (0.78 mL, 9.9 mmol, 2 equiv.) and 2-amino-4-(benzyloxy)-5-methoxyphenyl)(2-(hydroxymethyl)pyrrolidin-1-yl)methanone **S6** (175 mg, 4.95 mmol) in DCM (15 mL) at 0 °C. The reaction mixture was stirred for 10 minutes. H_2O and 1M HCl were added to the reaction mixture, which was then extracted with DCM (3 × 30 mL). The extracts were dried with $MgSO_4$ and concentrated *in vacuo*. Purification by flash chromatography (MeOH-$CHCl_3$ / 0.5:9.5 *v/v*) yielded **S7** as a yellow solid (1.16g, 64%). R_f = 0.32 (100% EtOAc); $[\alpha]^{20}_D$ = -82.29 (*c* = 0.48, $CHCl_3$); MS *m/z* 441.6 ($^+$); ^{1}H-NMR (400 MHz, $CDCl_3$) δ_H 8.72 (bs, 1 H), 7.88 (s, 1H), 7.48-7.46 (d, *J* = 7.4 Hz, 2 H), 7.39-7. 31 (m, 2 H), 6.84 (s, 1 H), 6.0-5.91 (m, 1 H), 5.38 -5.33 (dq, *J* = 17.1, 1.5 Hz, 1 H), 5.27- 5.23 (dq, *J* = 17.1 Hz, 1.4 Hz, 1 H), 5.17 (d, *J* = 2.5 Hz, 1 H), 4.65-4.62 (m, 1 H), 4.44 (d, *J* = 6.5 Hz, 1 H), 3.84 (s, 1 H), 3.76-3.66 (m, 1 H), 3.62-3.57 (m, 1 H), 3.53-46 (m, 1 H), 1.92-1.86 (m, 1 H), 1.77-1.64 (m, 2 H).

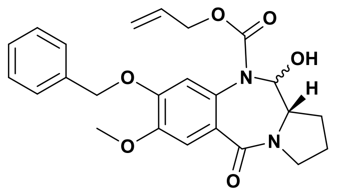

(11a*S*)-allyl 8-(benzyloxy)-11-hydroxy-7-methoxy-5-oxo-2,3,11,11a-tetrahydro-1*H*-benzo[e]pyrrolo[1,2-*a*][1,4]diazepine-10(5*H*)-carboxylate (S8).

TEMPO (2,2,6,6-tetramethyl-1-piperidinyloxy) (52 mg, 0.33 mmol, 1.5 equiv.) and DIAB (1 g, 0.33 mmol, 1.5 equiv.) were added to a solution of **S7** (980 mg, 0.22 mmol) in DCM (15 mL) and stirred overnight at room temperature. The organic layer was extracted with CH_2Cl_2 (15 mL), washed with sodium metabisulfite (2 × 20 mL), $NaHCO_3$ (2 × 20 mL), and brine (3 × 15 mL). The organic extracts were dried with $MgSO_4$ and evaporated under reduced pressure. Flash chromatography (EtOAc 100%) was carried out to give **S8** as a solid (520 mg, 53%). R_f = 0.25 (EtOAc); MS *m/z* 439.2 (M^+); ^{1}H-NMR (400 MHz, $CDCl_3$) δ_H 7.42-7.31 (m, 5H, Ar-H), 7.25 (s, 1H, H-9), 6.70 (s, 1H, H-9), 5.60 (d, 1H, *J* = 9.8 Hz), 5.17-5.06 (m, 4H), 4.47-4.43 (m, 2H), 4.12 (q, 1H, *J* = 7.0 Hz), 3.92 (s, 3H, OCH_3), 3.71-3.67 (m, 1H), 3.60-3.45 (m, 3H), 2.12-2.08 (m, 2H), 2.03-1.98 (m, 2H).

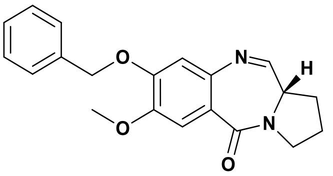

(*S*)-8-(benzyloxy)-7-methoxy-2,3-dihydro-1*H*-benzo[e]pyrrolo[1,2-*a*][1,4]diazepin-5(11a*H*)-one (15).

Pyrrolidine (114 µL, 1.35 mmol, 1.1 equiv.), PPh_3 (16 mg, 5 mol %) and then $Pd(PPh_3)_4$ (36 mg, 2.5 mol%) were added to a solution of **S8** (540 mg, 1.23 mmol) in CH_2Cl_2 (15 mL). The mixture was stirred at room temperature for 20 mins and then evaporated. The residue was purified by column chromatography (2% MeOH-EtOAc) to give **15** as a pale-yellow oil, which, after high vacuum solvent extraction, was recovered as a pale yellow solid (foam) (330 mg, 75%). M. p. = 78 - 82 ^{o}C. R_f = 0.33 (EtOAc:MeOH / 9.5:0.5 *v/v*); $[\alpha]^{20}_{D}$ = +661° (*c* = 0.20, $CHCl_3$); MS *m/z* 337.2 (M^+); ^{1}H-NMR (400 MHz, $CDCl_3$) δ_H 7.63 (d, *J* = 4.6 Hz, 1H), 7.53 (s, 1H, H-6), 7.45- 7.30 (m, 5H, Ar- H), 6.84 (s, 1H, H-9), 5.19 (q, *J* = 12.1, 9.5 Hz, 2H, $ArCH_2O$), 3.95 (s,3H), 3.83-3.77 (m, 1H, H-3). 3.72-3.68 (m, 1H, H-11a), 3.61-3.54 (m, 1H, H-3), 2.35- 2.27 (m, 2H, H-1), 2.06-2.02 (m, 2H, H-2).

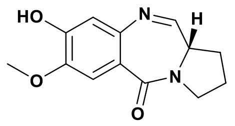

(*S*)-8-hydroxy-7-methoxy-2,3-dihydro-1*H*-benzo[e]pyrrolo[1,2-a][1,4]diazepin-5(11a*H*)-one (5).

To a solution of benzyl-protected PBD **10** (50 mg, 0.148 mmol) in DCM (5 mL) was added CH_2SO_3H (1 mL) at 0 °C. The reaction mixture was stirred for 10 mins at 0 °C then at room temperature for 2 hours. The solution was diluted with DCM, neutralised with cold 1 M $NaHCO_3$, extracted with CH_2Cl_2 and dried over $MgSO_4$. The reaction mixture was then evaporated under reduced pressure and purified by SiO_2 flash chromatography (CH_2Cl_2 then gradient 1-7% MeOH-CH_2Cl_2) to afford the title product **5** (7 mg, 19%). R_f = 0.21 (CH_2Cl_2-MeOH / 9.5:0.7 *v/v*); $[\alpha]^{20}_D$ = +207.14 (*c* = 1.17, $CHCl_3$); MS *m/z* 247.3 (M^+); ^{1}H-NMR (400 MHz, $CDCl_3$) δ_H 7.68-7.66 (d, 1H, *J* = 5.0 Hz, H-11); 7.53 (s, 1H, H-6), 6.89 (s, 1H, H-9), 3.96 (s, 3H, OCH_3), 3.75-3.70 (m, 1H, H-11a), 3.61-3.56 (m. 2H, H-3), 2.32-2.28 (m, 2H, H-1), 2.08-2.02 (m, 2H, H-2). ^{13}C-NMR (100 MHz, $CDCl_3$) δ_C 162.6 (C-11), 148.6, 148.3, 128.5, 112.5 (C-9), 111.1 (C-6), 56.1 (OCH_3), 46.73 (C-H11a), 29.6 (C-1), 24.1 (C-2).

The synthesis of 2-iodoadenosine (**11**)

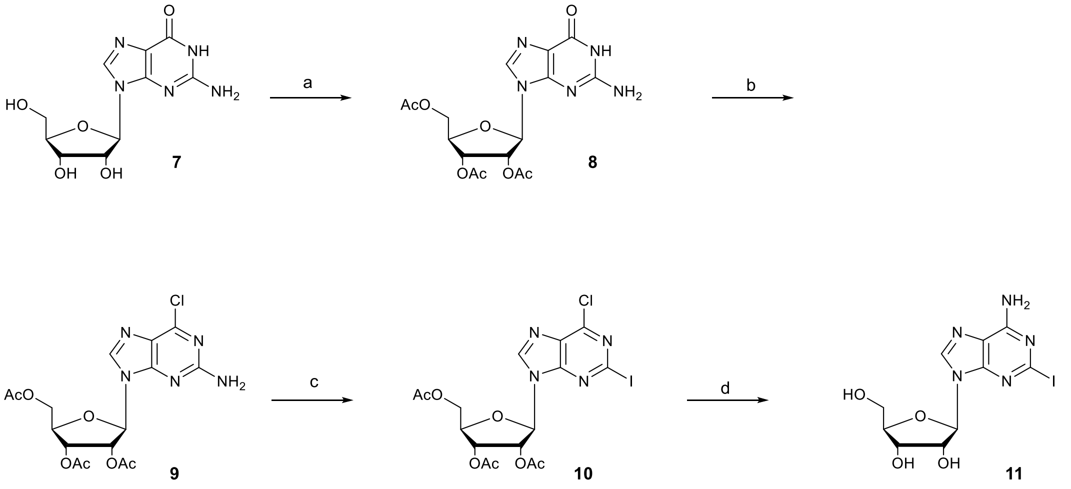

Scheme S2. *Reagents and conditions:* (a) Ac_2O, pyridine, DMF; (b) $POCl_3$, Et_4NCl, *N*,*N*,-dimethylaniline, acetonitrile; (c) CuI, I_2, CH_2I_2, isoamyl nitrite in THF, 80 °C; (d) NH_3/MeOH.

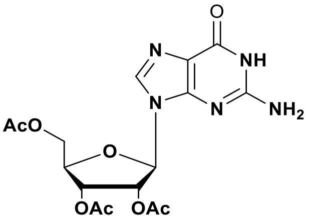

(2*R*,3*R*,4*R*,5*R*)-2-(acetoxymethyl)-5-(2-amino-6-oxo-1*H*-purin-9(6*H*)-yl)tetrahydrofuran-3,4-diyl diacetate (8).

A mixture of guanosine **7** (7.5 g, 26.5 mmol), which was dried in vacuo at 100 °C for 4 days over P_2O_5, acetic anhydride (15 mL), dry pyridine (7 mL), and dry DMF (30 mL) was heated at 75 °C and stirred for 3 h. The clear solution was filtered whilst hot, cooled to room temperature, and evaporated to produce a heavy crystalline suspension. The residue was treated with 30 mL of 2-propanol and filtered. The precipitate was washed with 2-propanol to give a white solid. The residue was treated with boiling 2-propanol with vigorous magnetic stirring. The partially dissolved suspension was stirred at reflux for 5 min and then was refrigerated at 5 ºC overnight. The product was filtered, washed with 2-propanol, and dried *in vacuo* at 80 °C, over P_2O_5, to give **8** as a white powder (8.4 g, 77%). M. p. = 229 - 231 ºC. Lit.[1] m. p. = 230 -233 ºC. R_f = 0.23 ($CHCl_3$-EtOH / 9:1 *v/v*) MS *m/z* 410.4 (M^+); ^{1}H-NMR (400 MHz, DMSO-*d6*) δ_H 10.79 (s, 1H, NH), 7.92 (s, 1H, H-8), 6.53 (br, 2H, NH_2), 5.98 (d, *J* = 6.1 Hz, 1H, H-1'), 5.79 (t, 1H, *J* = 6.0 Hz, H-4'), 5.49 (dd, *J* = 4.0, 1.6 Hz, 1H, H-3'), 4.39-4.23 (m, 3H, CH_2 and H-2'), 2.10 (s, 3H, OAc), 2.03 (d, 6H, *J* = 2.5 Hz, OAc).

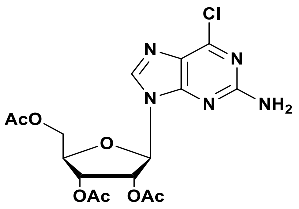

(2*R*,3*R*,4*R*,5*R*)-2-(acetoxymethyl)-5-(2-amino-6-chloro-9*H*-purin-9-yl)tetrahydrofuran-3,4-diyl diacetate (9).

To a round bottom flask were added compound **8** (8.4 g, 20.5 mmol) and Et_4NCl (6.8 g, 41 mmol, 2 equiv.), which were both pre-dried in *vacuo* at 85 °C overnight over P_2O_5, in freshly distilled acetonitrile (20 mL), and the solution was stirred at room temperature. Subsequently, distilled *N*,*N*-dimethylaniline (2.6 mL, 20.5 mmol, 1 equiv.) and phosphoryl chloride (11.6 mL, 123 mmol, 6 equiv.) were added to the stirred solution at room temperature. The flask was placed in a preheated hot plate at 100 °C and the solution was heated, whilst stirring at reflux, for 10 min. Volatile materials were evaporated *in vacuo*. The resulting yellow foam was dissolved in 100 mL of $CHCl_3$, and stirred vigorously with crushed ice for 15 min. The layers were separated and the aqueous phase was extracted with 5 × 35 mL of $CHCl_3$. The combined organic phase was kept cold by addition of crushed ice and was washed with 6 × 25 mL of cold water, 5% $NaHCO_3/H_2O$ to pH 7, dried over $MgSO_4$ for 1 h, and filtered. A 60 mL portion of 2-propanol was added and the combined filtrate was slowly evaporated *in vacuo*. The remaining residue solidified on cooling to room temperature. The residue was filtered, washed with 2-propanol (20 mL) and dried *in vacuo* overnight. The product was recrystallized from 150 mL of boiling 2-propanol yielding **9** as a solid (6.99 g, 79%). M. p. = 150 – 152 ^{o}C, Lit.[1] m. p. = 152 – 153 ^{o}C. R_f = 0.42 ($CHCl_3$:EtOH / 9.5:0.5 *v/v*); MS *m/z* 410.2 (M^+); ^{1}H-NMR (400 MHz, $CDCl_3$) δ_H 7.87 (s, 1H, H-8), 6.00 (d, *J* = 4.8 Hz, 1H, H-1'), 5.95 (t, *J* = 5.1 Hz, 1H, H-2' or H-4'), 5.74 (t, *J* = 5.0 Hz, 1H, H-2' or H-

4'), 5.22 (brs, 2H, NH_2), 4.47-4.36 (m, 3H), 2.14 (s, 3H, OAc), 2.09 (d, 6H, J = 6.6 Hz, 2 × OAc).

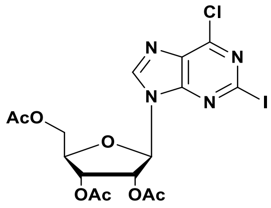

(2*R*,3*R*,4*R*,5*R*)-2-(acetoxymethyl)-5-(6-chloro-2-iodo-9*H*-purin-9-yl)tetrahydrofuran-3,4-diyl diacetate (10).

Isoamyl nitrite (6.63 mL, 49.2 mmol, 3 equiv.) was added to a mixture of **9** (6.99 g, 16.4 mmol), I_2 (4.14 g, 16.4 mmol, 1 equiv.), CH_2I_2 (13.17 mL, 17.22 mmol, 1.05 equiv.) and CuI (3.42 g, 17.96 mmol, 1.1 equiv.) in dry THF (60 mL). The reaction mixture was heated to reflux for 45 mins, cooled to room temperature, filtered and evaporated to dryness. Purification by flash chromatography ($CHCl_3$ then gradient 5% EtOH-$CHCl_3$) provided the desired compound **10** as a yellow foam (4.1 g, 47%). M. p. = 179 - 181 ^{o}C, Lit.[2] m. p. = 182 -183 ^{o}C. R_f = 0.21 ($CHCl_3$:EtOH 9.5:0.5 *v/v*); MS m/z 412.2 (M^+); ^{1}H-NMR (400 MHz, $CDCl_3$) δ_H 8.20 (1H, s), 6.20 (d, J = 5.6 Hz, 1H, H-8), 5.77 (t, J = 5.5 Hz, 1H), 5.58 (dd, J = 4.4 HZ, 1H), 4.50-4.47 (m, 1H), 4.42-4.40 (m, 2H), 2.17 (s, 3H, OAc), 2.14 (s, 3H, OAc), 2.10 (s, 3H, OAc); ^{13}C-NMR (100 MHz, $CDCl_3$) δ_C 170.4 (OAc), 169.7 (OAc), 169.6 (OAc), 152.2 (C-2), 151.3 (C-6), 143.3 (C-8), 132.5 (C-5), 117.2 (C-3), 86.9 (C-1'), 81.1 (C-2'), 73.6 (C-3'), 70.8 (C- 4'), 63.1 (CH_2), 21.3 (OAc CH_3), 20.7 (OAc CH_3), 20.6 (OAc CH_3).

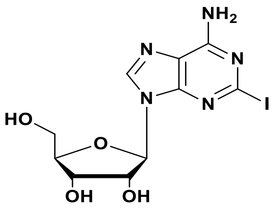

2-Iodoadenosine (11).

Compound **10** (0.10 g, 0.24 mmol) was added to 70% methanolic ammonia (3 mL) in a steel sealed tube and heated to 60 °C for 24 h. The tube was then cooled to room temperature and degassed, and the solvent was removed *in vacuo*. The residue was purified by flash chromatography ($CHCl_3$ then 20% EtOH-$CHCl_3$) to provide **11** as a white solid (36 mg, 49%). M. p. = 139 - 144 ^oC (softening from 105 °C, Lit.[2] m. p. = 145 -147 ^oC. R_f = 0.40 ($CHCl_3$:EtOH / 8:2 *v/v*); MS m/z 393.1 (M^+); ^{1}H-NMR (400 MHz, DMSO-d_6) δ_H 8.30 (s, 1H, H8), 7.72 (brs, 2H, NH_2), 5.80 (d, 1H, H1′, J = 6.2 Hz), 5.44 (d, J = 6.3 Hz, 1H, 2′-OH), 5.20 (d, J = 4.7 Hz, 1H, 3′-OH), 5.04 (t, J = 11.2 Hz, 1H, 5′-OH), 4.52 (dd, J = 5.8 Hz, 11.0 Hz), 4.11 (m, 1H) 3.93 (m, 1H′), 3.62–3.531 (m, 2H,); ^{13}C-NMR (100 MHz, DMSO-d_6) δ_C 155.9 (C-2), 149.8 (C-4), 139.4 (C-8), 87.1 (C-1’ or C-4’), 85.8 (C-1’ or C-4’), 73.5 (C-2’), 70.5 (C-3’), 61.4 (CH_2).

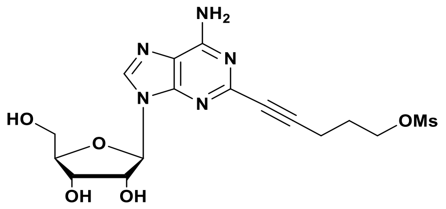

5-(6-amino-9-((2*R*,3*R*,4*S*,5*R*)-3,4-dihydroxy-5-(hydroxymethyl)tetrahydrofuran-2-yl)-9*H*-purin-2-yl)pent-4-yn-1-yl methanesulphonate (13).

To a stirred solution of 4-pentyn-1-ol (2.313 g, 3 mmol,) in CH_2Cl_2 (20 mL) was added triethylamine (5.4 mL, 3.9 mmol, 1.3 equiv.). Methanesulphonyl chloride (MsCl) (2.8 mL, 3.6 mmol, 1.2 equiv.) in DCM (5 mL) was then added dropwise over 30 mins at 0 °C. The reaction was warmed to room temperature and stirred for 1 h. The solution was washed with brine (30 mL) and the aqueous phase was extracted with CH_2Cl_2 (3 × 30 mL). The organic layer was dried and concentrated to dryness. Purification by flash chromatography (EtOAc-MeOH, 0-10%) gave the mesylated alkyne linker, which was used directly in the next step. MS *m/z* 163.2 (M^+). Pent-4-yn-1-yl methanesulfonate (350 mg, 2.16 mmol, 3.0 equiv.) was added dropwise via syringe pump over 0.5 h to a solution of 2-iodoadenosine (200 mg, 0.5 mmol, 1.0 equiv.), $Pd(PPh_3)_2Cl_2$ (35 mg, 0.051 mmol, 0.05 equiv.), and copper iodide (15 mg, 0.077 mmol, 0.076 equiv.) in CH_3CN:Et_3N (1:1 *v/v,* 10 mL). The reaction was stirred for 6 h at room temperature and then concentrated to dryness. Purification by flash chromatography (0–10% EtOAc-MeOH) afforded the title compound **13** (205 mg, 94%). R_f = 0.25 (EtOAc-MeOH 8:2, *v/v*); MS m/z 428 (M^+); ^{1}H-NMR (400 MHz, DMSO-d_6) δ_H 8.41 (s, 1H, H-8), 7.45 (s, 2H, NH_2), 5.85 (d, *J* = 6.3 Hz, 1H, H-1'), 5.43 (d, *J* = 4.6 Hz, 1H, H-2'), 4.52 (d, *J* = 4.2 Hz, 1H, H-3'),4.32- 4.29 (m, 2H, H-4'), 4.13 (d, *J* = 2.4 Hz, 2H, alkyl CH_2), 3.95 (dd, *J* = 7.9, 3.4 Hz, 1H), 3.69-3.66 (m, 2H, CH_2),

3.57-3.53 (m, 5H, CH_2 and 3 × OH), 3.18 (s, 3H, CH_3). 2.31 (m, 2H, alkyl CH_2), 2.01 (t, 2H, alkyl CH_2).

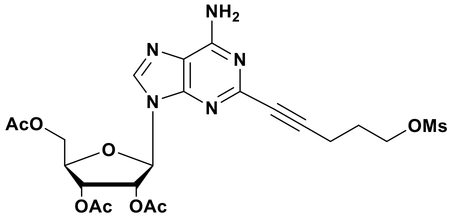

(2*R*,3*R*,4*R*,5*R*)-2-(acetoxymethyl)-5-(6-amino-2-iodo-9*H*-purin-9-yl)tetrahydrofuran-3,4-diyl diacetate (14).

Triethylamine (0.8 mL, 5.7 mmol) was added to a suspension of **13** (205 mg, 0.46 mmol), DMAP (15 mg, 0.12 mmol, 0.27 equiv.), and Ac_2O (0.7 mL, 6.86 mmol) in CH_3CN (20 mL). The mixture was stirred for 1 h at room temperature then MeOH (1 mL) was then added. The solution was concentrated to dryness and the residue was partitioned between EtOAc and H_2O. The separated organic phase was dried over $MgSO_4$ and concentrated to dryness to give **14** (196 mg, 74%). R_f = 0.5 (EtOAc:MeOH / 8:2, *v/v*); MS m/z 554.5 (M^+). ^{1}H-NMR (400 MHz, CD_3OD) δ_H 8.26 (s, 1H, H-8), 6.29 (d, *J* = 3.9 Hz, 1H, H-1'), 6.00 (t, *J* = 6.3 Hz 1H, H-4'), 5.81-5.74 (td, *J* = 14.3, 5.2 Hz, 1H, H-3'), 4.47-4.44 (m, 4H, CH_2OMs, CH_2OAc, H-2'), 3.12 (s, 3H, CH_3), 2.70-2.61 (td, *J* = 20.9, 10.1 Hz, 2H, CH_2), 2.1 (s, 3H, OAc), 2.08-2.05 (m, 6H, 2 × OAc), 1.10 (t, *J* = 7.2 Hz, 2H, alkyl CH_2).

References

1. Robins, M. J.; Uznański, B. *Can. J Chem.* **1981**, 59, 2601-2607.

2. Matsuda, A.; Shinozaki, M.; Yamaguchi, T.; Homma, H.; Nomoto, R.; Miyasaka, T.; Watanabe, Y.; Abiru, T. *J Med Chem.* **1992**, 35, 241-252.

Appendix I

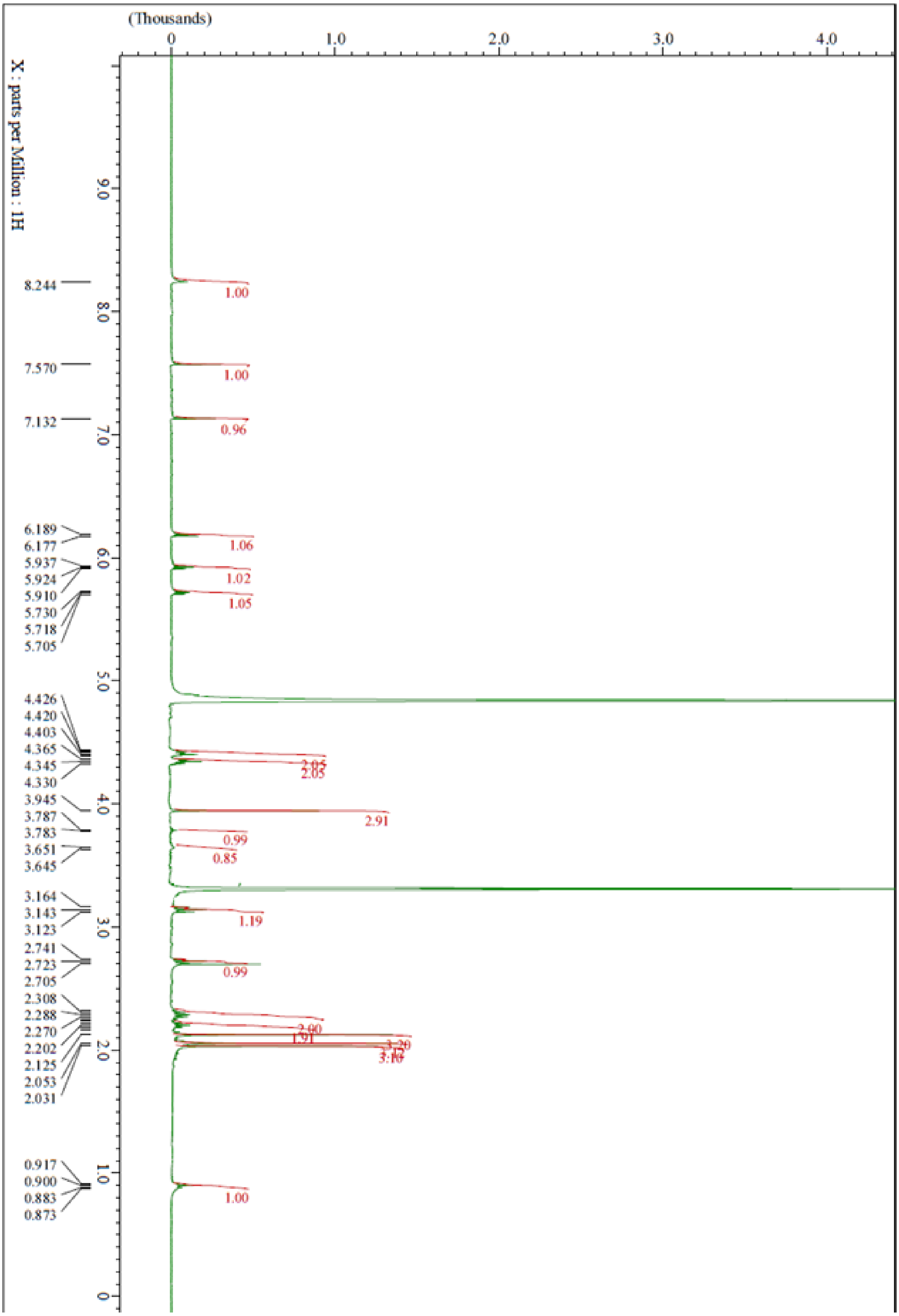

(Thousands)
0
1.0
2.0
3.0
4.0
X : parts per Million : 1H
9.0
8.0
7.0
6.0
5.0
4.0
3.0
2.0
1.0
0
8.244
7.570
7.132
6.189
6.177
5.937
5.924
5.910
5.730
5.718
5.705
4.426
4.420
4.403
4.365
4.345
4.330
3.945
3.787
3.783
3.651
3.645
3.164
3.143
3.123
2.741
2.723
2.705
2.308
2.288
2.270
2.202
2.125
2.053
2.031
0.917
0.900
0.883
0.873
1.00
1.00
0.96
1.06
1.02
1.05
2.05
2.91
0.99
0.85
1.19
0.99
1.00

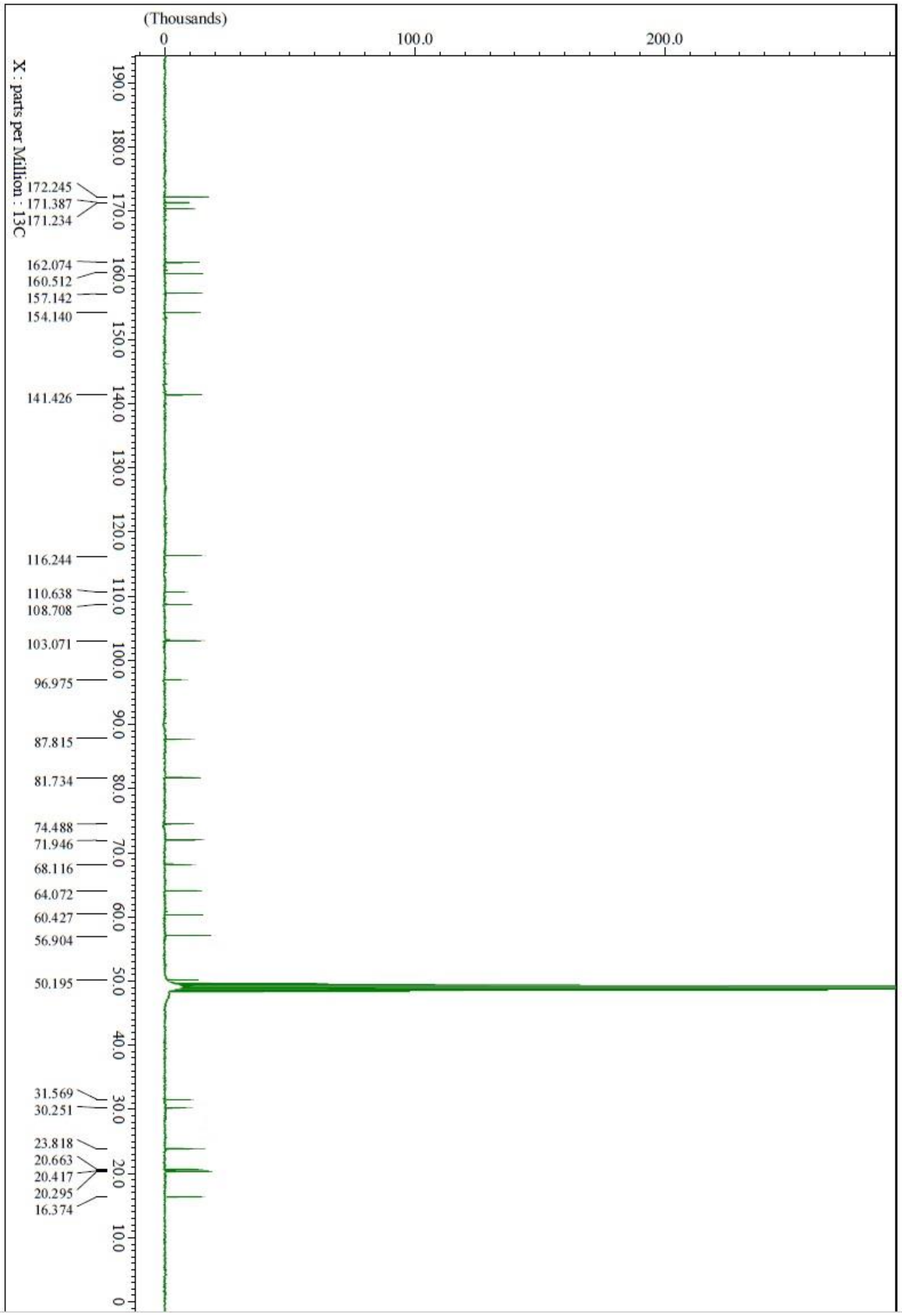
(Thousands)
0
100.0
200.0
X : parts per Million : 13C
172.245
171.387
171.234
162.074
160.512
157.142
154.140
141.426
116.244
110.638
108.708
103.071
96.975
87.815
81.734
74.488
71.946
68.116
64.072
60.427
56.904
50.195
31.569
30.251
23.818
20.663
20.417
20.295
16.374
190.0
180.0
170.0
160.0
150.0
140.0
130.0
120.0
110.0
100.0
90.0
80.0
70.0
60.0
50.0
40.0
30.0
20.0
10.0
0
